# Copper Metabolism in *Naegleria gruberi* and Its Deadly Relative *Naegleria fowleri*

**DOI:** 10.3389/fcell.2022.853463

**Published:** 2022-04-11

**Authors:** Kateřina Ženíšková, Maria Grechnikova, Robert Sutak

**Affiliations:** Department of Parasitology, Faculty of Science, Charles University, BIOCEV, Vestec, Prague, Czechia

**Keywords:** copper, alternative oxidase, alternative NADH dehydrogenase, *Naegleria gruberi*, *Naegleria fowleri*, DJ-1, CTR copper transporters, electron transport chain

## Abstract

Although copper is an essential nutrient crucial for many biological processes, an excessive concentration can be toxic and lead to cell death. The metabolism of this two-faced metal must be strictly regulated at the cell level. In this study, we investigated copper homeostasis in two related unicellular organisms: nonpathogenic *Naegleria gruberi* and the “brain-eating amoeba” *Naegleria fowleri*. We identified and confirmed the function of their specific copper transporters securing the main pathway of copper acquisition. Adjusting to different environments with varying copper levels during the life cycle of these organisms requires various metabolic adaptations. Using comparative proteomic analyses, measuring oxygen consumption, and enzymatic determination of NADH dehydrogenase, we showed that both amoebas respond to copper deprivation by upregulating the components of the branched electron transport chain: the alternative oxidase and alternative NADH dehydrogenase. Interestingly, analysis of iron acquisition indicated that this system is copper-dependent in *N. gruberi* but not in its pathogenic relative. Importantly, we identified a potential key protein of copper metabolism of *N. gruberi*, the homolog of human DJ-1 protein, which is known to be linked to Parkinson’s disease. Altogether, our study reveals the mechanisms underlying copper metabolism in the model amoeba *N. gruberi* and the fatal pathogen *N. fowleri* and highlights the differences between the two amoebas.

## Introduction

Transition metals are required in many crucial biological processes of all living organisms. The most abundant redox-active metal in cells is iron, which is followed by other metals such as copper, manganese, cobalt, molybdenum, and nickel (Andreini et al., 2008). Both iron and copper are crucial for the survival of organisms; however, excess concentrations of these metals can be toxic: iron can catalyze the generation of free radicals through the Fenton reaction, causing cellular damage, while copper probably binds to proteins and replaces iron from iron-sulfur cluster-containing proteins, impairing their function and causing iron-induced toxicity (Macomber and Imlay, 2009; Festa and Thiele, 2012; García-Santamarina and Thiele, 2015). On the other hand, copper is a cofactor of at least 30 cuproenzymes with a wide variety of roles, such as electron transport in respiration (cytochrome c oxidase CCOX) or free radical detoxification (superoxide dismutase SOD) (Solomon et al., 2014), and its necessity for biological systems arises from its ability to cycle between two redox states, Cu^1+^ and Cu^2+^, all of which contribute to organisms needing to evolve mechanisms to strictly regulate intracellular levels of these potentially harmful metals. Homeostatic mechanisms consist of their uptake, transport, storage, and detoxification pathways. Interestingly, the metabolism of iron and copper are linked, which is best demonstrated by the copper dependence of iron acquisition by the cell, as was shown in yeasts with FET3 multicopper oxidase in the high-affinity iron uptake system (Askwith et al., 1994). The best-described mechanism of copper acquisition involves high-affinity copper transporters named Ctrs. Their function and structure are widely conserved from yeast to humans (Nose et al., 2006; Maryon et al., 2007).

Very little is known about copper transporters in parasitic protists. In *Plasmodium falciparum*, a copper efflux P-ATPase has been identified and partly characterized (Rasoloson et al., 2004), as was a copper-binding protein with sequence features characteristic of copper transporters, including three transmembrane domains: an extracellular copper-binding methionine motif and transmembrane Gx_3_G and Mx_3_M motifs (Choveaux et al., 2012). Copper transporting ATPases were also identified in trypanosomatid parasites (Isah et al., 2020; Paul et al., 2021). Significantly more research has been performed on copper metabolism in pathogenic yeasts. Ctr homologs responsible for cellular copper uptake were identified in *Candida albicans* (CTR1) (Marvin et al., 2003) and in *Cryptococcus neoformans* (CTR1 and CTR4) (Sun et al., 2014). Importantly, homologs of these proteins are present in the genomes of both amoebas used in our study, *Naegleria gruberi* and *Naegleria fowleri* (Fritz-Laylin et al., 2010; Liechti et al., 2019).

*N. gruberi* and *N. fowleri* are unicellular organisms living worldwide in freshwater environments (De Jonckheere, 2004; Mull et al., 2013). *N. gruberi* is considered to be the best safe system to study the pathogenic “brain-eating amoeba” *N. fowleri*, which can infect people and cause primary amoebic meningoencephalitis (PAM), a rare but almost always fatal disease (Mungroo et al., 2021). The genomes of these organisms suggest canonical aerobic metabolism, such as the employment of cytochromes and ubiquinone in the respiratory chain, as well as properties of anaerobic metabolism, such as Fe-hydrogenase (Tsaousis et al., 2014), which is typically utilized in metabolic processes of organisms adapted to anaerobic conditions (Fritz-Laylin et al., 2011; Herman et al., 2021). Both amoebas were recently shown to be able to adjust their metabolism to reflect iron availability, downregulating nonessential and predominantly cytosolic iron-dependent pathways and utilizing available iron primarily in mitochondria to maintain essential energy metabolism (Mach et al., 2018; Arbon et al., 2020). In our previous study, we described how *N. fowler*i handles copper toxicity by upregulating a specific copper-exporting ATPase, a key protein of the copper detoxification pathway (Grechnikova et al., 2020). Recent study has found that *Cryptococcus neoformans* is able to sense different Cu environment during infection: high Cu in lungs and low Cu level in brain and is able to adapt its Cu acquisition in these different niches (Sun et al., 2014). Consequently, we focused the current study on the effect of copper deficiency on the metabolism of the brain-eating amoeba as well as its related nonpathogenic model amoeba *N. gruberi*. Herein, we show the role of Ctr homologs identified in both amoebas by functional complementation of mutant yeast lacking high-affinity copper transporters and demonstrate the effect of copper availability on several important components of cell proteomes, iron acquisition, and respiration in both amoebas. We demonstrate that both amoebas can reflect copper-limited conditions by upregulating parts of the respiratory chain to maintain maximal cell respiration. *N. gruberi* adapts to copper-limited conditions by inducing alternative oxidase, similar to the mechanism described in *C. albicans* (Broxton and Culotta, 2016), while *N. fowleri* upregulates alternative NDH-2 dehydrogenase. Moreover, we identified the potential key protein of copper metabolism in *N. gruberi*, the homolog of the DJ-1 protein.

## Materials and Methods

### Identification of *Naegleria* CTRs

*Naegleria* CTR genes were found by BLAST in the genomes of *N. fowleri* in the AmoebaDB database (Amos et al., 2021) and *N. gruberi* in the JGI PhycoCosm database (Grigoriev et al., 2021) using *Saccharomyces cerevisiae* CTR1 (YPR124 W), CTR2 (YHR175 W), and CTR3 (YHR175 W) gene sequences. Two predicted CTRs of *N. fowleri* (NF0078940, NF0118930) and three predicted CTRs of *N. gruberi* (gene IDs: NAEGRDRAFT_61759, NAEGRDRAFT_61987, and NAEGRDRAFT_62836) were identified.

### Functional Complementation Spot Assay of Predicted Ctrs of *N. gruberi* and *N. fowleri*

To synthesize *N. gruberi* and *N. fowleri* cDNA, SuperScript™ III reverse transcriptase (Thermo Fisher Scientific, United States) was used according to the manufacturer’s protocol. CTR genes were amplified from cDNA using a Q5 (NEB, United States) and Pfu DNA polymerase mixture (Promega, United States). The resulting products were subcloned into a pUG35 plasmid with a GFP tag (Güldener and Hegemann, http://mips.gsf.de/proj/yeast/info/tools/hegemann/gfp.html) and a pCM189 plasmid (Garí et al., 1997) with a tetracycline-regulatable promotor. The yeast mutant strain ctr1Δ/ctr3Δ (kindly provided by Dennis J. Thiele, Duke University, Durham, North Carolina) was transformed with pCM189 plasmids containing one of the predicted CTRs from *N. fowleri* (NF0078940, NF0118930) or *N. gruberi* (NAEGRDRAFT_61759, NAEGRDRAFT_61987, NAEGRDRAFT_62836). To observe the effect of complementation on phenotype, transformed yeasts were grown overnight in liquid SC-ura medium with 2% glucose (complete synthetic medium without uracil) at 30°C. Cells were diluted to an OD_600_ of 0.2, and 5-µl aliquots of four serial 10-fold dilutions were spotted onto SC-ura plates containing 2% raffinose as a carbon source.

### Localization of Ctrs by Fluorescence Microscopy

For protein localization, wild-type (WT) yeast BY4741 cells were transformed with pUG35 containing either the NgCTR or NfCTR gene, and transformants were grown overnight at 30°C in liquid SC-ura medium, washed and resuspended in phosphate-buffered saline (PBS), pipetted onto a microscope slide and mixed with the same volume of 2% agarose. The microscope slide was then covered with a cover slide and sealed. A fluorescent signal was detected using a Leica TCS SP8 WLL SMD confocal microscope (Leica, Germany) with an HC PL APO CS2 63x/1.20 water objective, excited at 488 nm and detected within 498–551 nm by a HyD SMD detector. The PMT detector was used for bright-field imaging. The resulting images were processed by LAS X imaging software (Leica Microsystems, Germany) and ImageJ (Schneider et al., 2012). Yeast transformation was performed according to a previously published protocol (Gietz and Schiestl, 2007).

### Amoeba Cultivation

#### Organisms

a) *N. gruberi* strain NEG-M, which was kindly provided by Lilian Fritz-Laylin (University of Massachusetts Amherst, United States), was grown axenically at 27°C in M7 medium (Fulton, 1974) with the addition of penicillin (100 U/ml) and streptomycin (100 μg/ml) in a 25-cm^2^ aerobic cultivation flask.
b) Axenic culture of *N. fowleri* strain HB-1, which was kindly provided by Dr. Hana Peckova (Institute of Parasitology, Biology Center CAS), was maintained at 37°C in 2% Bactocasitone (Difco) medium supplemented with 10% heat-inactivated fetal bovine serum (Thermo Fisher Scientific) with the addition of penicillin (100 U/ml) and streptomycin (100 μg/ml).

#### Cultivation Conditions

a) For comparative proteomic analysis, oxygen consumption measurements, measurement of the enzyme activity of complex I and NDH-2, and preparation of the samples for SDS–PAGE, copper deprivation was achieved by incubation of cells for 72 h in the presence of 5 µM neocuproine or 25 µM bathocuproinedisulfonic acid disodium salt (BCS), while copper enrichment was achieved by the addition of 25 µM Cu_2_SO_4_.
b) To examine the effect of copper availability on NgDJ-1 expression by western blot, cells were grown in 25 µM BCS or 1 μM, 25 μM, or 750 µM Cu_2_SO_4_ for 72 h.
c) The localization of NgDJ-1 was determined by western blotting of crude fractions of *N. gruberi* cultivated without the addition of copper or chelators and by fluorescence microscopy of cells cultivated for 72 h with 100 µM Cu_2_SO_4_ or 25 µM BCS.
d) To investigate the effect of ROS on NgDJ-1 expression, *N. gruberi* cells were preincubated in 10 µM rotenone or 20 µM PEITEC for 24 h, and cells with no addition of ROS-inducing agents were used as a control.

### Crude Fractionation of *N. gruberi* and *N. fowleri* Cells

The grown cells were washed twice in S-M buffer (250 mM Saccharose, 10 mM MOPS, pH 7.2) and disrupted by sonication using SONOPULS ultrasonic homogenizer mini20 (BANDELIN, Germany) with the following settings: amplitude 30%, 1/1 s pulse for 1 min on ice. Disrupted cells were evaluated under a microscope, and sonication was repeated until most of the cells were disrupted. The samples were then centrifuged for 10 min at 1,200 g and 4°C. To obtain the mitochondria-enriched fraction, the supernatant was centrifuged at 14,000 g for 20 min at 4°C. The pellet was used as a mitochondrial-enriched fraction and diluted to the same protein concentration as the supernatant (cytosol-enriched fraction).

### The Effect of Different Copper Chelators on the Growth of *N. gruberi* and *N. fowleri*

To investigate the effect of copper deprivation on the growth of *N. gruberi* and *N. fowleri*, the cultures were grown in the presence of copper chelators BCS (concentrations: 25 and 100 µM) and neocuproine (concentrations: 5 and 20 µM). Copper-rich conditions (25 µM Cu_2_SO_4_) were used as a control. Since BCS binds copper extracellularly and consequently its effect may only be evident after a longer period, we observed the effect of this chelator in long-term growth analysis with a dilution of the cells after 2 days. Each condition was set up in three independent biological replicates with starting culture concentrations of 50 000 cell/ml (*N. gruberi*) and 5,000 cell/ml (*N. fowleri*), and the cell concentration was measured every day by a Cell Counter (Beckman Coulter, United States). The effect of the intracellular copper chelator neocuproine was observed only at one time point: 72 h. Cells were grown in three biological replicates with starting concentrations of 1 × 10^4^ cells/ml (*N. gruberi*) and 4 × 10^3^ cells/ml (*N. fowleri*), and the cell concentration was measured on a Guava easyCyte 8HT flow cytometer (Merck, Germany) after treatment with 2% paraformaldehyde.

### ICP–MS Analysis

Cultures of *N. gruberi* and *N. fowleri* were grown in triplicate for each condition, washed three times (1,200 g, 10 min, 4°C) in 10 mM HEPES with 140 mM NaCl buffer, pH 7.2, and pelleted by centrifugation. The pellets were dried at 100°C, digested in 65% HNO_3_ in Savillex vials overnight at room temperature, incubated for 2 h at 130°C in Savillex vials (Millipore, United States) and diluted to a final volume of 10 ml in deionized water. The copper concentration was determined by inductively coupled plasma–mass spectrometry (ICP–MS) using iCAP Q ICP–MS (Thermo Fisher Scientific).

### LC–MS

*N. gruberi* and *N. fowleri* cells were grown in biological triplicates for each condition. After incubation, cells were pelleted by centrifugation (1,200 g, 10 min, 4°C) and washed three times with phosphate-buffered saline (PBS). Whole-cell label-free proteomic analysis followed by the same method as that described in (Mach et al., 2018) was performed by applying nanoflow liquid chromatography (LC) coupled with mass spectrometry (MS). Data were evaluated with MaxQuant software (Cox et al., 2014) using the AmoebaDB *N. fowleri* database downloaded in August 2018 or the *N. gruberi* database (downloaded from UniProt August 2018). Selected proteins (lacking annotation) were manually annotated using HHpred (Zimmermann et al., 2018) or NCBI BLAST (Altschul et al., 1990). The resulting data were further processed by Perseus software (Tyanova et al., 2016). Potential contaminants and reverse hits were filtered out. To evaluate significantly changed proteins at the level of the false discovery rate, Student’s t test with Benjamini–Hochberg correction was used. Proteins that were significantly changed and those found in only one condition (in at least two of three replicates) were selected. Proteins identified by only one peptide and proteins with Q-value higher than 0 were excluded from the selection.

### RT–qPCR

*Naegleria* cells were grown in copper-rich and copper-deficient conditions, and control untreated cells were grown for 72 h in quadruplicate. Cells were washed twice with PBS and spun (1,200 g, 4°C, 10 min). Total RNA was isolated using the High Pure RNA Isolation Kit (Roche, Switzerland). The KAPA SYBR^®^ FAST One-Step universal kit (Sigma Aldrich, United States) was used for RT–PCR according to the manufacturer’s protocol. RT–PCR was performed on a RotorGene 3000 PCR cycler (Corbett Research, Australia) under the following conditions: 42°C for 30 min (reverse transcription), 95°C for 5 min, and 40 cycles of 95°C for 10 s, 55°C for 20 s, and 72°C for 20 s; for melt-curve analysis, the temperature change was set from 55 to 95°C with a 1°C step and 5 s per step. The abundance of transcripts was calculated after normalization to the endogenous reference gene β-actin.

### Obtaining the NgDJ-1 Recombinant Protein for Antibody Preparation

The sequence of NgDJ-1 (XP_002680488.1) was obtained from the UniProt database (in August 2019), and bioinformatic analysis was performed by InterProScan in Geneious Prime^®^ 2019 2.3 (www.geneious.com), including protein domain prediction software such as Phobius (Käll et al., 2004), Pfam (Mistry et al., 2021), PANTHER (Thomas et al., 2003), and SignalP 5.0 (Almagro Armenteros et al., 2019) (see Supplementary Figure S4). The NgDJ-1 gene was amplified from cDNA without the transmembrane domain at the N-terminal part of the NgDJ-1 gene (primers: forward 5′-CAC​CAT​ATG​GTC​GAG​GCT​CAG​AAT​ATT​GAT​CAC-3′, reverse 5′-CAC​GGA​TCC​ATT​TTG​CTT​ATT​CAA​GAG​CTT​GT-3′) and subcloned into the vector pET42b (Merck) containing the C-terminal histidine tag. The protein was expressed in *E. coli* BL21 (DE3) (Merck) induced by 0.5 mM isopropyl β-D-1-thiogalactopyranoside (IPTG, Sigma Aldrich) for 4 h at 37°C. Protein was purified under denaturing conditions according to the manufacturer’s protocol using Ni-NTA agarose beads (Qiagen, Germany).

### NfNDH-2 and NgDJ-1 Antibody Production

NgDJ-1 and NfNDH-2 polyclonal antibodies were produced by David Biotechnology (Germany) in rabbits. NgDJ-1 antibody was prepared using purified HIS-tagged recombinant protein, while NfNDH-2 was prepared by 3 synthesized immunogenic peptides preselected by David Biotechnology (HDRQVSFAKSIHKPNEKKN, HEDYHYFEGKAIAIDTENQR, DPKSKKILVTDHLKVKGFE). To obtain a more specific signal, the produced antibodies NgDJ-1 and NfNDH-2 were purified by the SulfoLink Immobilization Kit for Proteins (Thermo Fisher Scientific) or the AminoLink Plus Immobilization Kit (Thermo Fisher Scientific), respectively. All purification procedures were performed following the manufacturer’s manual.

### Sample Preparation for SDS–PAGE, Native PAGE, and Western Blot

Cells were grown under specific conditions for 72 h, washed two times with PBS, pelleted at 1,000 g, 10 min, 4°C, and diluted to equal protein concentration determined by BCA Protein Assay Kit (Sigma Aldrich). Denatured samples (100°C for 5 min) were separated by SDS electrophoresis, blotted onto a nitrocellulose membrane (Amersham Protram 0.2 μm PC, GE Healthcare Life Sciences, United States), and incubated with specific polyclonal antibody at the following concentrations: anti-AOX (Agrisera, Sweden) 1:100, anti-NgDJ-1 1 1:50, and anti-NfNDH-2 1:1,000. HRP-conjugated goat anti-rabbit or anti-mouse antibodies (BioRad, United States) were used as secondary antibodies. Antibodies were detected using Immobilon Forte Western HRP substrate (Merck) on an Amersham Imager 600 (GE Health care Life Sciences, United States).

Crude fractions of *N. gruberi* (Chapter 2.5) used for localization of NgDJ-1 by western blot were prepared the same as SDS samples described above, but crude fractions of *N. fowleri* (Chapter 2.5) used for localization of NfNDH-2 were treated with 1% digitonin (Sigma Aldrich), incubated for 5 min on ice and resuspended in ×5 native sample buffer. The samples were then loaded on a native gel (containing 0.1% Triton TX-100, Sigma Aldrich) and separated by PAGE under native conditions.

### Immunofluorescence Microscopy

*N. gruberi* cells were stained with 100 nM MitoTracker Red CMXRos (Thermo Fisher Scientific) for 30 min in M7 medium in the dark at 27°C. After incubation, the medium was exchanged, and the cells were fixed with 1% formaldehyde for another 30 min. The treated cells were then carefully centrifuged (800 g, 5 min, 24°C), resuspended in PEM (100 mM piperazine-N,N′-bis(2-ethane sulfonic acid), 1 mM ethylene glycol-bis(β-aminoethyl ether)-N,N,N′,N′-tetraacetic acid, and 0.2 mM MgSO_4_) and transferred onto cover slides. The cell slides were then incubated for 1 h in PEMBALG blocking solution (1% BSA, 0.1% NaN_3_, 100 mM L-lysin, 0.5% cold water fish skin gelatin in PEM). The NgDJ-1 protein was visualized by an anti-rat antibody coupled to Alexa Fluor 488 (Thermo Fisher Scientific) (dilution 1:1,000) bound to a custom-made rat polyclonal antibody (dilution 1:100). Slides with stained cells were mounted by Vectashield with DAPI (Vector Laboratories, United States). The signal was detected by a TCS SP8 WLL SMD confocal microscope (Leica) equipped with an HC PL APO CS2 63x/1.20 oil objective, excited by 509 nm and detected within 526–655 nm by a HyD SMD detector. The PMT detector was used for bright-field imaging and processed as described in Chapter 2.3.

*N. fowleri* microscopy slides for visualization of NfNDH-2 were prepared as described above except that *N. fowleri* cells were immobilized on slides covered with poly-L-lysine and all the staining, including 10 nM MitoTracker Red CMXRos and primary and secondary antibodies, were completed on slides rather than in a cultivation flask.

### Measurements of Oxygen Consumption

*N. fowleri* and *N. gruberi* cells were grown for 72 h in biological triplicates or tetraplicates, respectively, under copper-depleted and copper-rich conditions, pelleted (1,200 g, 10 min, 4°C), washed twice, and resuspended to the same cell concentration in glucose buffer (50 mM glucose, 0.5 mM MgCl_2_, 0.3 mM CaCl_2_, 5.1 µM KH_2_PO_4_, 3 µM Na_2_HPO_4_, pH 7.4). The cell concentration was measured on a Guava easyCyte 8HT flow cytometer (Merck). Cell respiration in each sample was measured by detecting oxygen decreases using the Clark-type electrode system Oxygen meter Model 782 (Strathkelvin) in a Mitocell Mt 200 cuvette in a total volume of 700 µl. The whole system was calibrated for 27°C for *N. gruberi* and 37°C for *N. fowleri*. Specific inhibitors of alternative oxidase, salicyl hydroxamic acid (SHAM) at concentrations of 0.5 mM (*N. gruberi*) or 0.2 mM (*N. fowleri*), and an inhibitor of complex IV, KCN, at concentrations of 2.4 mM (*N. gruberi*) or 2.05 mM (*N. fowleri*) were used. The protein concentration of the sample was determined using a BCA kit (Sigma Aldrich).

### Complex I and NDH-2 Enzyme Activity of *Naegleria fowleri*

Total NADH dehydrogenase activity was measured by the following protocol. Three biological replicates of *N. fowleri* were cultivated for 72 h in copper-rich or copper-deficient conditions. The cells were spun down, washed with S-M buffer (250 mM saccharose, 10 mM MOPS, pH 7.2), and diluted to the same cell concentration. Next, the cells were treated with 1% digitonin (Sigma Aldrich) for 5 min on ice and added to a reaction mixture containing 100 µM KPi buffer at pH 7.5 and 300 µM NADH. The reaction was started by the addition of 50 µM nonnatural coenzyme Q2 (in 99.9% ethanol, Sigma Aldrich), an analog of Q10. The activity was measured for 5 min at 340 nM wavelength on a Shimadzu UV-2600 UV–VIS spectrophotometer (Shimadzu, Japan) with UV Probe software (Shimadzu).

The activity of alternative NADH dehydrogenase (NDH-2) was estimated as the remaining activity measured in digitonine-treated culture preincubated for 5 min with the inhibitor of Complex I, 75 µM rotenone. The activity of complex I was calculated as the remaining activity after NDH-2 activity subtraction from overall NADH dehydrogenase activity. The protein concentration was determined by a BCA kit (Sigma Aldrich).

### Iron Uptake

*N. fowleri* and *N. gruberi* cells were grown in copper-rich and copper-deficient conditions for 72 h, pelleted (1,200 g, 10 min, 4°C), washed twice, resuspended in glucose-HEPES medium (50 mM glucose, 20 mM HEPES, pH 7.2) and diluted to the same cell concentration of 1 × 10^6^ cells per sample. The cell concentration was estimated by a Guava easyCyte 8HT flow cytometer (Merck). Samples were incubated for 1 h with 1 µM ^55^Fe-citrate or with 1 µM ^55^Fe-citrate with the addition of 1 m mM ascorbate to reduce iron to the ferrous form. Uptake was stopped by the addition of 1 mM EDTA, and the cells were then washed three times with glucose-HEPES buffer, diluted to the same protein concentration [using a BCA kit (Sigma Aldrich)] and separated using the Novex Native PAGE Bis-Tris Gel system (4–16%, Invitrogen, United States). The gel was dried for 2 h in a vacuum and autoradiographed by Typhoon FLA 7000 (GE Life Sciences, United States) using a tritium storage phosphor screen (GE Life Sciences).

## Results

### Identification of Copper Uptake Proteins (Ctrs) of *N. gruberi* and *N. fowleri*

**FIGURE 1:**
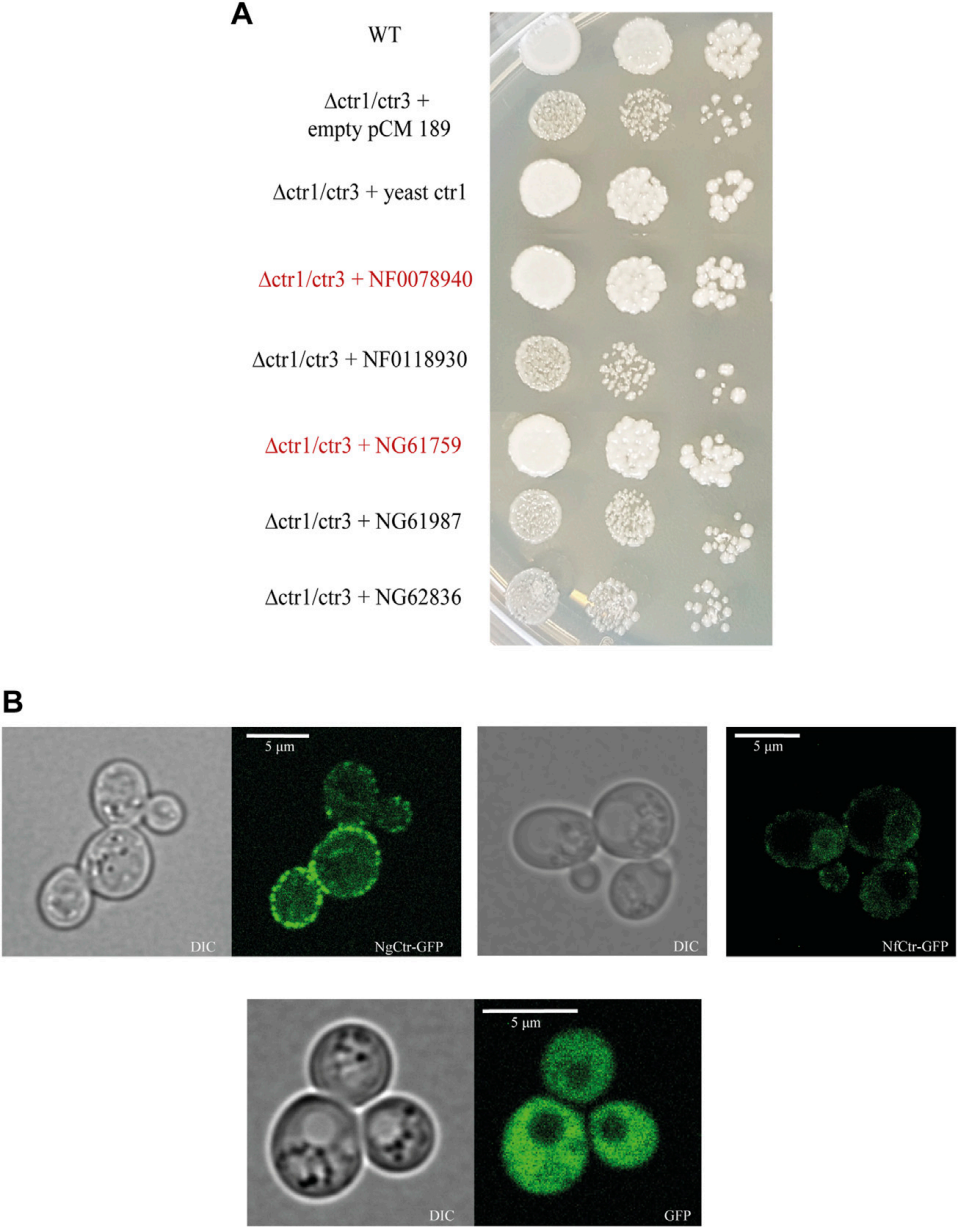
Identification of copper uptake proteins (Ctrs) of *Naegleria gruberi* and *Naegleria fowleri.* **(A)** Functional complementation spot assay of NgCTRs and NfCTRs in Δctr1/ctr3 mutant yeast cells lacking genes for high-affinity copper transporters. Wild-type BY4741 cells transformed with the empty tetracycline-regulated expression vector pCM189 and Δctr1/ctr3 mutant cells transformed with the same plasmid carrying yeast CTR1, NfCTRs (NF0078940, NF0118930) or NgCTRs (NG61759, NG61987, NG62836) were spotted onto synthetic complete medium without uracil with 2% raffinose as the carbon source and grown for 72 h at 30°C. Mutant yeast cells with plasmids containing CTR genes from *N. fowleri* (NF0078940) and *N. gruberi* (NG61759), which functionally complement the missing yeast copper transporters CTR1 and CTR3, are marked in red. **(B)** Localization of NfCtr (NF0078940) and NgCtr (NG61759) by fluorescence microscopy. Wild-type yeast BY4147 expressing copper transporters linked with GFP from *N. gruberi* NgCTR1-GFP (NG61759 + pUG35), *N. fowleri* NfCTR1-GFP (NF0078940 + pUG35), and GFP (empty pUG35 vector).

In the genomes of both amoebas, we identified several homologs of copper transporters based on a BLAST search using *S. cerevisiae* CTR1, CTR2, and CTR3. To confirm the copper uptake function of these candidate transporters, we performed a functional complementation assay using copper transporter 1 and copper transporter 3 double knockout yeast strain (ctr1Δ/ctr3Δ). One of the selected Ctrs from each amoeba (NgCTR1, NAEGRDRAFT_61759, and NfCTR1, NF0078940) restored copper transporter function in the yeast mutant and showed typical localization to the yeast plasma membrane (Figure 1A and Figure 1B). The localization of the other homologs is shown in Supplementary Figure S1. To determine whether *N. fowleri* and *N. gruberi* regulate the copper acquisition pathway depending on the availability of the metal at the transcriptional level, as shown in *S. cerevisiae* (Winge et al., 1998), we analyzed the abundance of CTR transcripts by RT–PCR in cells grown under low copper and copper-rich conditions. Our data showed no significant copper-induced changes in the RNA levels of the selected CTR genes (NF0078940, NF0118930*,* NAEGRDRAFT_61759, NAEGRDRAFT_61987, NAEGRDRAFT_62836) (Supplementary Figure S2). This result is not unexpected considering our previous study, where we found that iron starvation-induced changes in *N. fowleri* were mostly posttranslational (Arbon et al., 2020)**.**

### Comparative Proteomic Analysis Revealed That ETC Components and NgDJ-1 Are the Most Affected by Copper Deprivation

Since *Naegleria* is not prone to genetic manipulations, we chose whole-cell label-free comparative proteomics to gain complex insight into the metabolic adaptation to copper limitations. To elucidate the effect of copper deprivation on the viability of *N. gruberi* and *N. fowleri* cells, we cultivated both amoebas for 72 h in the presence of the copper chelators bathocuproinedisulphonic acid (BCS) and neocuproine (Neo). Both chelators are selective for Cu^+^, and in contrast to the extracellular copper chelator BCS, neocuproine can chelate copper intracellularly.

**FIGURE 2:**
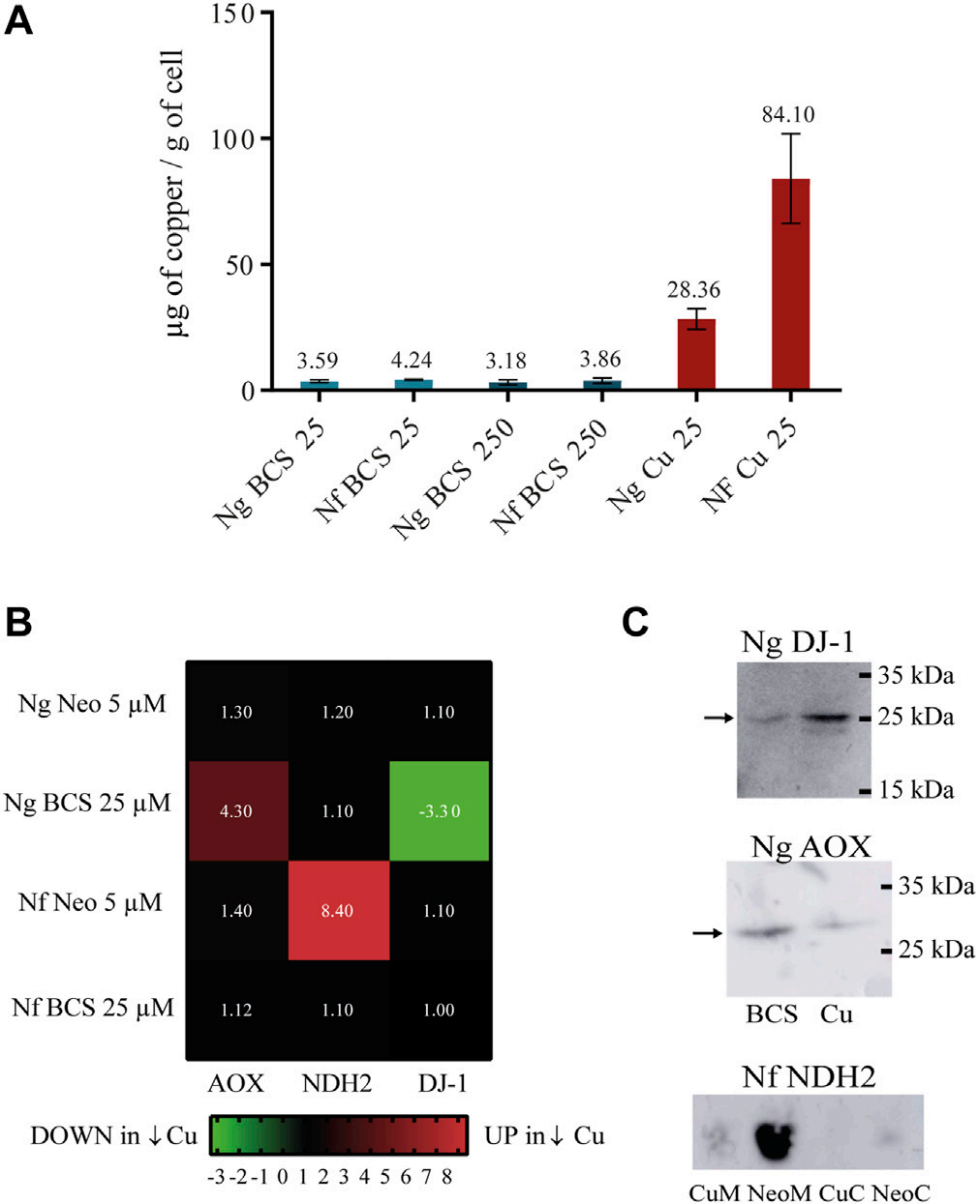
Effect of copper availability on *Naegleria gruberi* and *Naegleria fowleri*. **(A)** Copper contents in *N. gruberi* and *N. fowleri* supplemented with 25 µM Cu_2_SO_4_, 25 μM BCS, or 250 µM BCS for 72 h. The values represent the mean of copper content of three individual replicates. **(B)** The fold changes of the most relevant proteins in this article are demonstrated on the heatmap; upregulation under copper-limited conditions is indicated by red color and downregulation by green color. The heatmap demonstrates that the chelators have different effects on both amoebas. **(C)** western blot analysis confirming the proteomic results using the specific antibodies antiNgDJ-1, antiNfNDH2, and antiAOX on cell lysates of *N. gruberi* incubated with 25 µM BCS (BCS) or 25 µM Cu_2_SO_4_ (Cu) and separated by SDS–PAGE and on cytosolic (C) and mitochondrial (M) fractions of *N. fowleri* cells incubated with 25 µM Cu_2_SO_4_ (Cu) or 5 µM neocuproine (Neo) separated by clear native PAGE.

To determine the amount of copper within the cells grown in the presence of BCS, we analyzed those samples using ICP–MS. Figure 2A shows that this extracellular copper chelator causes a significant decrease in copper accumulation compared to control (cells cultivated in presence of 25 µM Cu_2_SO_4_) after 72 h, while the effect is not further increased using a 10-fold higher concentration. The effect of BCS on copper accumulation was more pronounced in *N. fowleri*, which has a higher overall intracellular amount of copper under control conditions; however, the growth of *N. fowleri*, unlike *N. gruberi*, was not affected by this chelator (Supplementary Figure S3). This indicates that *N. fowleri* possesses more efficient copper homeostasis mechanisms than its nonpathogenic relative, and the use of an intracellular chelator is required to observe the physiological response to copper starvation. Based on these results, we decided to perform two proteomic analyses using the cells grown with the addition of 25 µM BCS and the cells grown in 5 µM neocuproine to achieve copper deprivation for both amoebas. To ensure sufficient copper status, the cells cultivated in presence of 25 µM Cu_2_SO_4_ were used as a control sample for both proteomic analyses. This concentration was used based on our previous work where we elucidated the IC50 of copper being 1 mM for *N. gruberi* and as high as 1.6 mM for *N. fowleri* (Grechnikova et al., 2020).

The list of all proteins that were significantly changed under copper deprivation is presented in Supplementary Table S1. In the analysis of the resulting proteomic data, we focused on proteins that are likely to bind copper or are related to copper metabolism, potentially compensating for the lack of copper or being involved in the oxidative stress response or energy metabolism. The selected proteins meeting these criteria are listed in Table 1. A heatmap presenting the most relevant proteins identified in this study demonstrates that selected chelators cause different effects on amoebas (Figure 2B).

**TABLE 1:** Selected *N. gruberi* and *N. fowleri* proteins whose abundance was significantly changed under copper-limited conditions in at least one condition and in one amoeba. Arrows indicate significant upregulation or downregulation, and no arrow sign in proteins with a fold change lower than 1.5 indicates no significant change.

*Naegleria gruberi*			
**Fold Change in BCS**	**Fold Change in Neo**	**Accession Number in Database**	**Database Annotation/Manual Annotation**
↑4.3	1.3	XP_002681229.1	AOX
1.1	1.2	XP_002672148.1	NDH-2
↓3.3	1.1	XP_002680488.1	DJ-1
↑2.0	Not Found	XP_002680302.1	Hemerythrin
1.4	↓1.9	XP_002674924.1	Thioredoxin reductase
↓2.2	1.2	XP_002670102.1	Glutathione-S-transferase
↓1.6	1.3	XP_002670607.1	Glutathione-S-transferase III
***Naegleria fowleri***			
**Fold change in BCS**	**Fold change in Neo**	**Accession Number in Database**	**Database Annotation/Manual Annotation**
1.1	1.4	NF0004720	AOX
1.1	↑8.4	NF0090420	NDH-2
1	1.1	NF0125230	DJ-1
1.4	↑3.5	NF0127030	Hemerythrin
1.0	1.2	NF0014440	Thioredoxin reductase
1.1	1.3	NF0101120	Glutathione-S-transferase
1.1	Not Found	NF0101840	Glutathione-S-transferase
1.1	1.1	NF0039660	Glutathione-S-transferase

The mitochondrial electron transport chain of *Naegleria gruberi* and *Naegleria fowleri* consists of complexes I, II, and III, two terminal oxidases: alternative oxidase (AOX) and cytochrome c oxidase (complex IV), and an alternative NADH ubiquinone oxidoreductase. Our proteomic analysis revealed that both amoebas responded to copper starvation by upregulating alternative enzymes involved in the electron transport chain. AOX (XP_002681229.1) from *N. gruberi* showed a 4.3-fold upregulation under copper-limited conditions. In contrast to *N. gruberi, N. fowleri* reacted to copper starvation through 8.4-fold upregulation of the protein NF0090420 (partial sequence) identified as nonproton pumping alternative NADH dehydrogenase (NDH-2). The complete sequence of this protein was obtained from genome of *N. fowleri* strain ATCC 30894 (AmoebaDB - FDP41_010952) (Supplementary Figure S4).

Additionally, the proteins involved in reactive oxygen species (ROS) detoxification pathways were significantly downregulated in copper-limited *N. gruberi* (thioredoxin reductase and two homologs of glutathione-S-transferase). Furthermore, in both amoebas, we observed significant upregulation in the expression of hemerythrin under copper limitation, a protein that probably plays a role in the defense against oxidative stress in bacteria (Li X. et al., 2015; Ma et al., 2018). Together, these results indicate that copper deprivation in naeglerias may lead to the generation of ROS.

Interestingly, one of the most downregulated proteins (fold change 3.3) of *N. gruberi* in copper-limited conditions was protein XP_002680488.1, which is a homolog of DJ-1 family proteins (Supplementary Figure S4). These proteins are thought to perform many functions (Bandyopadhyay and Cookson, 2004; Wei et al., 2007). Some studies on the human homolog of DJ-1 claim its ability to bind copper and serve as a copper chaperone for Cu, Zn superoxide dismutase (Girotto et al., 2014).

The copper-induced changes in protein abundance were also confirmed using western blot analysis with specific antibodies (Figure 2C). The localization of NfNDH-2 by fluorescence microscopy to demonstrate the antibody specificity is shown in Supplementary Figure S5.

### RT qPCR Analysis Revealed That Changes Caused by Copper Deprivation Are Posttranslational

Selected genes encoding copper-regulated proteins (NfNDH-2, NfHemerythrin) were also analyzed by RT qPCR using copper-starved and control cells. Transcript abundance was normalized to the endogenous reference gene β-actin. Analogous to CTRs, no changes in the transcriptional level of these selected genes were observed, suggesting that the proteomic response of both amoebas to copper starvation occurs at the posttranslational level (Supplementary Figure S2).

### The Activity of NgAOX and NfNADH-2 Reflects Copper Availability

**FIGURE 3:**
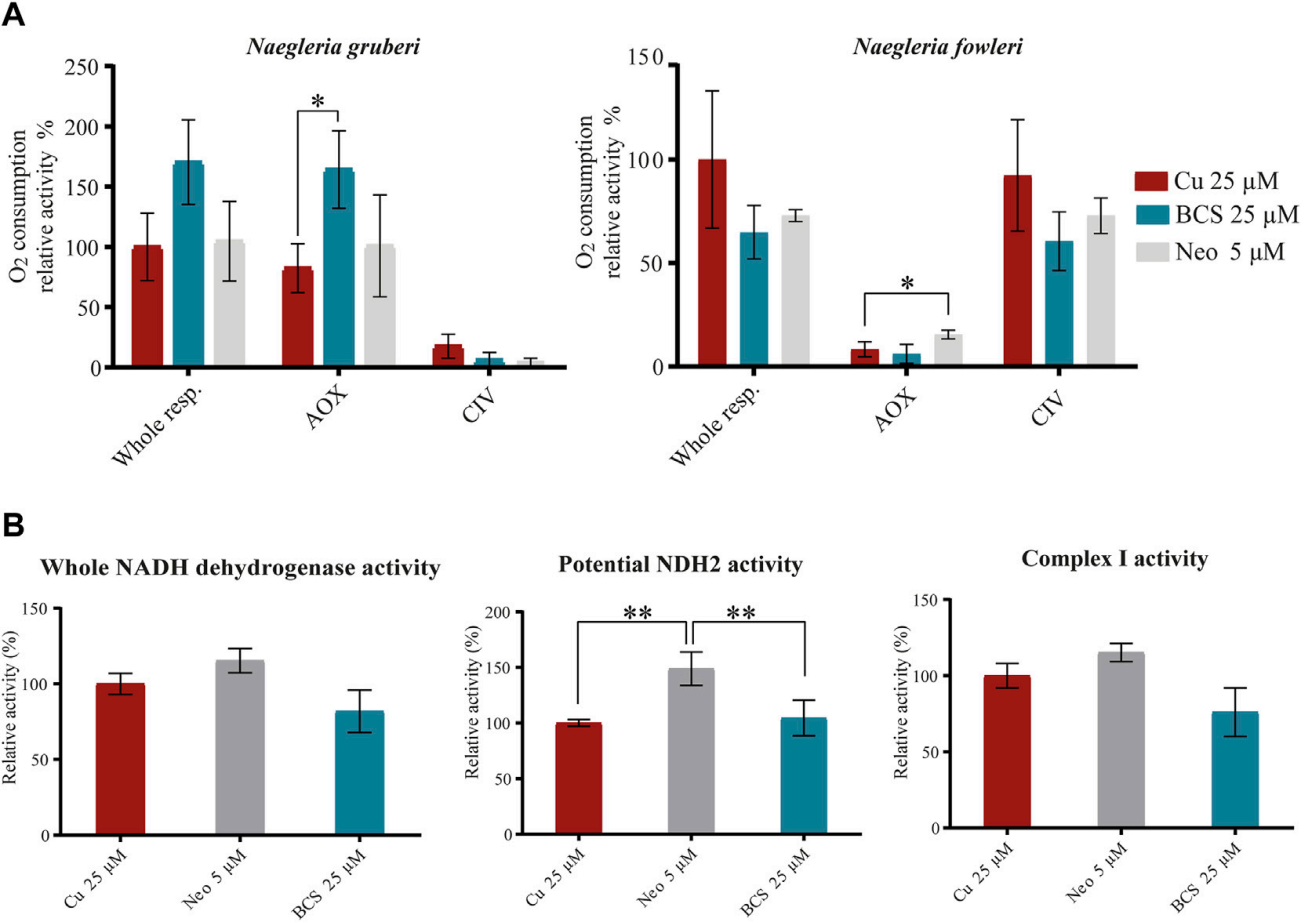
The activity of NgAOX and NfNDH2 reflects the proteomic analysis results. **(A)** Oxygen consumption of NgAOX is increased due to copper deprivation. Oxygen consumption was measured in *N. gruberi* and *N. fowleri* cells incubated for 72 h with 25 M BCS or 5 µM neocuproine, and control cells were supplemented with 25 µM Cu_2_SO_4_. Specific inhibitors of alternative oxidase (SHAM) and complex IV (KCN) were used to distinguish the activities of these enzymes. For easier interpretation, oxygen consumption activity was calculated as the relative activity, where the whole respiration of control samples (Cu 25 µM) corresponds to 100%. The graphs show the mean of three biological replicates with the standard deviation. To evaluate the statistical confidence, Student’s t test was used (* indicates a *p* value ≤0.05). **(B)** The enzyme activity of NfNDH2 is increased under low copper conditions. Spectrophotometric measurement of NADH dehydrogenase activity was measured using nonnatural Q2, the analog of Q10, at 340 nm on *N. fowleri* cells incubated in low or normal copper conditions. The potential NDH2 activity was measured after preincubation of the cells with rotenone. The activity of complex I was calculated by subtracting the potential NDH2 activity from overall NADH dehydrogenase activity. For easier interpretation, the activities are shown as the relative activity (%), and the activities of control cells (Cu 25 µM) correspond to 100%. To evaluate the statistical confidence, Student’s t test was used (**indicates a *p* value ≤0.01).

Our proteomic analysis indicated rearrangement of the mitochondrial electron transport chain in both amoebas under copper starvation. To determine the physiological effect of copper availability on respiration, we performed an oxygen consumption assay with amoebas grown in copper-limited conditions. Both organisms possess two terminal oxidases, cytochrome c oxidase (CCOX) and alternative oxidase (AOX), which couple the electron flow from ubiquinol with the reduction of O_2_ to H_2_O (Cantoni et al., 2020). In contrast with CCOX, AOX does not participate in ATP generation. The activity of NgAOX corresponded to the proteomic results: in copper-limited *N. gruberi,* the activity of AOX was almost twice as high as that in control cells (Figure 3A). In *N. fowleri*, copper starvation did not result in the upregulation of alternative oxidase at the protein level, but its activity in neocuproine-treated cells was significantly increased (Figure 3A). Interestingly, our results also demonstrate that *N. gruberi* respiration is predominantly mediated by alternative oxidases, whereas *N. fowleri* respires mainly through complex IV (Figure 3A).

In addition to the classical rotenone-sensitive NADH dehydrogenase, the electron transport chain of both amoebas additionally contains an alternative rotenone-insensitive NADH dehydrogenase (NDH-2). These enzymes share the same functions, but NDH-2 does not contribute to the generation of the transmembrane proton gradient. The comparative proteomic analysis indicated that *N. fowleri* adapts to copper-deprived conditions by upregulation of NDH-2. The resistance of NDH-2 to rotenone was used to distinguish this enzyme from classical rotenone-sensitive NADH dehydrogenase. When NADH dehydrogenase activity of lysates of control and copper-limited cells were compared, the rotenone-sensitive complex I activity was not affected by copper, while the rotenone-resistant activity was higher in the neocuproine-treated cells than in the control sample. Although we cannot exclude that other enzymes contribute to this activity, considering the proteomic data, we believe that the main enzyme responsible for the measured activity is NfNDH-2 (Figure 3B).

### Expression of the Mitochondrially Localized Protein NgDJ-1 is Copper-dependent and is Not Induced by ROS Accumulation

**FIGURE 4:**
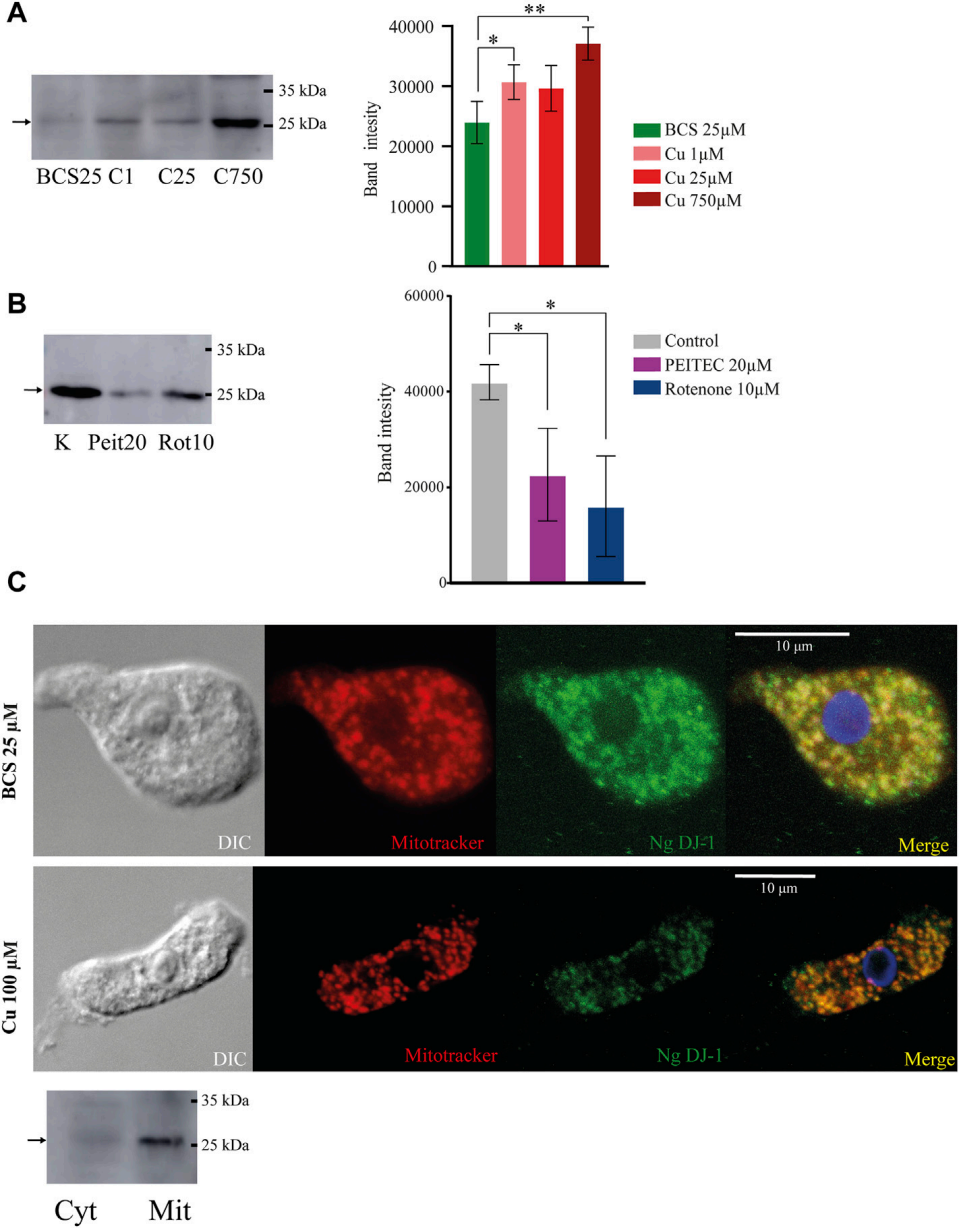
The expression of mitochondrially localized NgDJ-1 is copper-dependent and is not induced by ROS accumulation. **(A)** NgDJ-1 protein expression is copper-dependent. Western blot analysis of whole-cell lysates of cells incubated for 72 h in low or high copper conditions (BCS 25 µM and Cu_2_SO_4_ 1/25/750 µM) using an anti-NgDJ-1 antibody. To compare signal strength in each condition, densitometry of four independent replicates was performed using ImageJ. Changes in signals are demonstrated on the graph showing the mean of the signal with standard deviation error bars (*indicates a *p* value ≤0.05; **indicates a *p* value ≤0.01. **(B)** ROS accumulation does not induce NgDJ-1 expression. Western blot analysis of DJ-1 expression in cells preincubated with the ROS-inducing agents 20 µM PEITC and 10 µM rotenone for 24 h. The control sample (K) was *N. gruberi* without any additions. The graph demonstrates the difference in signal strength in each condition. **(C)** Localization of NgDJ-1 is mitochondrial regardless of copper availability. NgDJ-1 was visualized by immunofluorescence microscopy using a polyclonal antibody (anti-NgDJ-1) on *N. gruberi* cells precultivated with 25 µM BCS and 100 µM Cu_2_SO_4_ for 72 h. MitoTracker CMXRos (Thermo Fisher) was used for visualization of mitochondria. DIC–differential interference contrast. Mitochondrial localization of NgDJ-1 was also demonstrated by immunoblot detection of cytosolic (Cyt) and mitochondria-enriched (Mit) fractions of *N. gruberi*.

One of the most downregulated proteins in copper-deprived *N. gruberi* cells (3.3-fold change downregulation in BCS) is protein XP_002680488.1 (named NgDJ-1 in this article), which shows homology to proteins belonging to the DJ-1/ThiJ/PfpI superfamily (Supplementary Figure S4). This superfamily contains functionally and structurally diverse proteins, many of which remain only poorly characterized at the biochemical level (Bandyopadhyay and Cookson, 2004). To confirm the connection between copper availability and NgDJ-1 expression, we performed a western blot analysis of whole-cell lysates of *N. gruberi* grown in copper-deprived conditions as well as in media supplemented with copper at different concentrations (1 μM, 25 μM, and 750 µM). The results demonstrate that the copper-induced expression of DJ-1 observed in our proteomic analysis is even more pronounced when cells are exposed to copper at levels that probably lead to toxicity, indicating a role of this protein in copper metabolism (Figure 4A).

The human homolog of DJ-1 has many predicted functions but is mainly annotated as a redox sensor and ROS scavenger (Zhang et al., 2020). Considering this, we analyzed the lysates of *N. gruberi* cells exposed for 24 h to two ROS-inducing agents, rotenone and PEITC, by western blot using an NgDJ-1 antibody. Unexpectedly, we observed that treatment with both agents resulted in a decrease in NgDJ-1 expression (Figure 4B).

To determine NgDJ-1 cellular localization, we used two different methods: fluorescence microscopy and western blot analysis of crude cell fractions. Both methods revealed mitochondrial localization of NgDJ-1, which is rather unusual for this protein (Figure 4C). Predictably, observed molecular weight of DJ-1 on western blots is lower than anticipated (∼26 kDa instead of 30 kDa), probably due to cleavage of mitochondrial targeting sequence. More pictures are shown in (Supplementary Figure S6).

### *N. gruberi* Iron Uptake is Copper-Regulated

**FIGURE 5:**
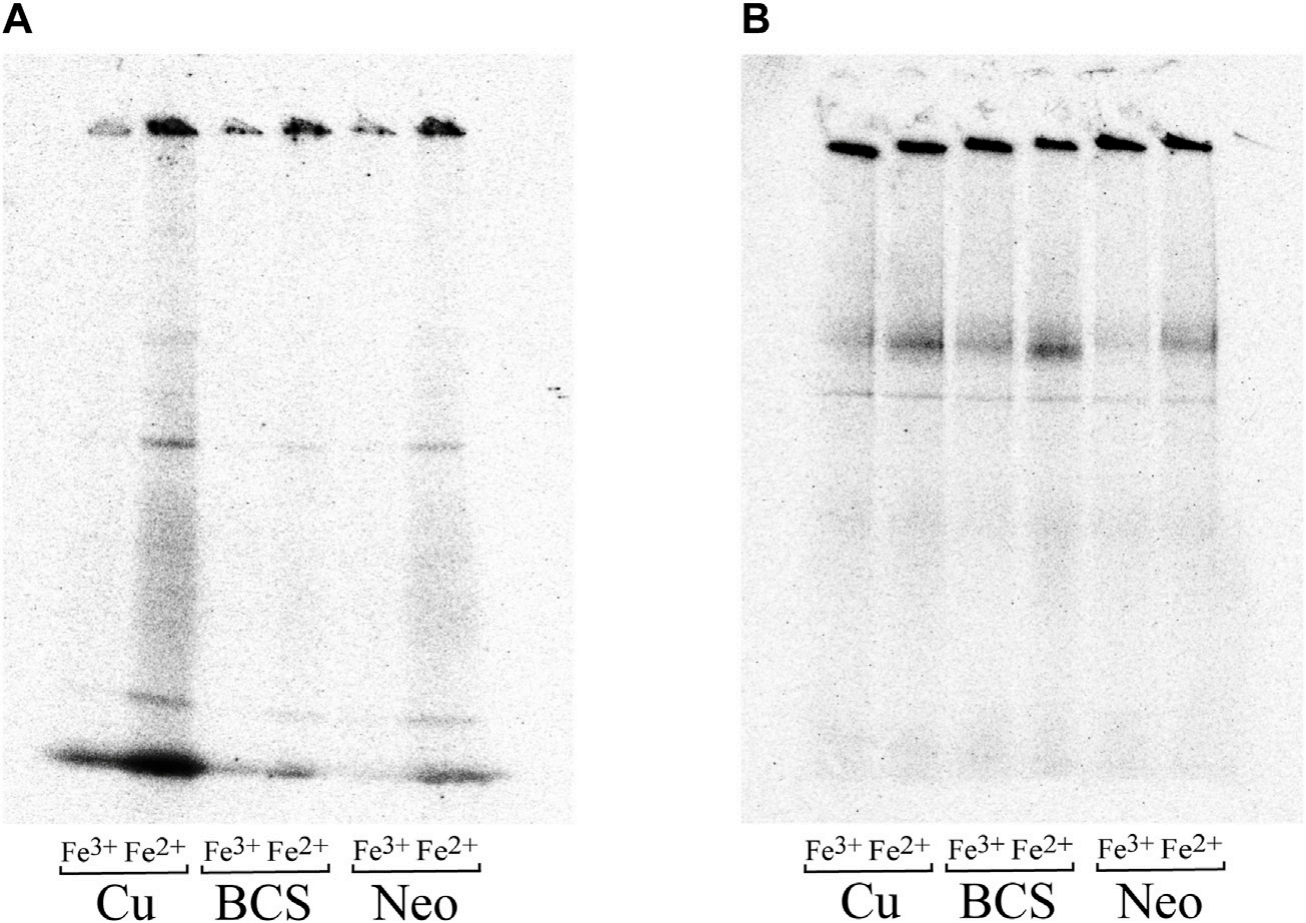
Iron uptake is regulated by copper in *N. gruberi* but not in *N. fowleri.* Ferrous and ferric iron uptake by *N. gruberi* **(A)** and *N. fowleri* **(B)** under copper-rich and copper-deficient conditions. Autoradiography of blue native electrophoresis gels of whole-cell lysates of *N. gruberi* and *N. fowleri* cells incubated with 25 µM BCS (BCS), 5 µM neocuproine (Neo), and 25 µM Cu_2_SO_4_ (Cu) for 72 h and further incubated with ^55^Fe(II) (ferrous ascorbate) or ^55^Fe(III) (ferric citrate) for 1 h.

Iron uptake mechanisms in various organisms are frequently interconnected with copper. To determine this connection in both amoebas, we employed blue native electrophoresis analysis allowing visualization of the incorporation of iron radionuclide into cellular protein complexes. In our previous work, we showed that *N. fowleri* prefers to take up the reduced form of iron (Fe^II^) and that iron acquisition is not induced by iron starvation (Arbon et al., 2020). Here, we show that similarly, the nonpathogenic relative *N. gruberi* preferably acquired a reduced form of iron, indicating a reductive uptake mechanism. Importantly, our results demonstrate that iron uptake is significantly affected by copper availability (Figure 5A), suggesting the requirement of copper in some of the iron acquisition system components (e.g., multicopper oxidase). In contrast, iron uptake efficiency in *N. fowleri* remained unaffected by copper deprivation (Figure 5B).

## Discussion

### Copper and host:pathogen Interface

An important host defense process called nutritional immunity occurs at the host-pathogen interface: the host restricts access to essential metals for the pathogen (Hood and Skaar, 2012). Iron sequestration during bacterial infection is well described, and bacterial pathogens have developed a variety of strategies to circumvent host-mediated iron limitation (Weinberg, 1975; Posey and Gherardini, 2000; Cassat and Skaar, 2013). In contrast to iron, copper is usually utilized in the opposite manner by host immune cells, which use the toxic properties of this metal to kill pathogens (Festa and Thiele, 2012; Hodgkinson and Petris, 2012; Stafford et al., 2013; Chaturvedi and Henderson, 2014). However, in fungal infections caused by *C. neoformans* and *C. albicans,* the pathogens experience limited copper availability in the host in specific situations. Although host immunity employs the toxic properties of copper in the lungs, which are the main locations of *C. neoformans* infection (Ding et al., 2013), *C. neoformans* tends to disseminate to the brain in immunodeficient hosts, where copper may be restricted. *C. neoformans* adapts to these copper-limited conditions by inducing specific copper uptake transporters (Waterman et al., 2007; Sun et al., 2014). *C. albicans* occupies diverse niches inside the host, but it may disseminate through the bloodstream, and the major location of infection in the murine model is the kidney. In the early stage of kidney infection, copper levels increase briefly, but as the infection progresses, the level of copper drops. *C. albicans* responds to decreasing copper conditions by switching from copper-dependent Cu/Zn SOD to Mn SOD3 and, interestingly, by upregulating alternative oxidase (AOX) to minimize mitochondrial damage and simultaneously maximize COX respiration (Li CX. et al., 2015; Besold et al., 2016; Broxton and Culotta, 2016).

In a recent study, we described how *N. fowleri* handles the toxic properties of copper and identified the key protein of the copper detoxification pathway, a copper-translocating ATPase (Grechnikova et al., 2020). In the present study, we focused on other aspects of copper metabolism and aimed to elucidate the metabolic adaptations of the free-living unicellular organism *N. gruberi* and its pathogenic relative *N. fowleri* to low copper availability.

### Copper Acquisition

Our study began with a search for proteins involved in copper acquisition by both amoebas. In eukaryotic cells, the import of copper to the cytoplasm is widely mediated by high-affinity copper transporter (Ctr) localized to the plasma membrane. Ctr is an integral membrane protein conserved from yeast to humans with high specificity for Cu(I) (Zhou and Gitschier, 1997; Kozlowski et al., 2009). In *Saccharomyces cerevisiae*, copper is transported into cells by two high-affinity transporters, CTR1 (Dancis et al., 1994b) and CTR3 (Knight et al., 1996), and a low-affinity copper transporter, CTR2, is responsible for the mobilization of vacuolar copper ions (Rees et al., 2004; Liu et al., 2012). All CTRs of *S. cerevisiae* are regulated by intracellular copper status (Jungmann et al., 1993; Winge et al., 1998). We identified genes encoding potential Ctrs in the genomes of both amoebas, including three genes in *N. gruberi,* and two in *N. fowleri.* Since effective genetic manipulation of these organisms has not been established, we decided to verify copper transport function by expression in yeasts and by functional complementation assay using the ctr1Δ/ctr3Δ mutant yeast strain. One of the proposed CTRs from each amoeba was able to restore copper import function and showed typical localization to the plasma membrane. In contrast to *S. cerevisiae*, neither CTR appears to be regulated by copper starvation at the transcriptional level in *Naegleria*, indicating that the amoebas do not respond to copper starvation at the copper acquisition level or that the regulation is posttranslational.

### Branched Mitochondrial ETC

Our proteomic approach to understanding the metabolic adaptations to copper limitation yielded particularly interesting findings: some of the proteins comprising the electron-transporting chain (ETC), the key part of the energy metabolism of a cell, are among the most affected by low copper availability in both amoebas. The ETC of both Naeglerias is branched and, in addition to the classical arrangement of complexes (CI-IV), possesses two nonenergy-conserving components: cyanide insensitive alternative oxidase (AOX) and alternative NADH dehydrogenase (NDH-2), both of which are significantly upregulated in copper-deprived conditions. Branched mitochondrial ETC is also known from plants, fungi, and other protists, some of which are human pathogens (e.g., *C. albicans*, *C. neoformans*, *Acanthamoeba castellanii*). AOX bypasses complex III and complex IV, but its activity is not coupled to proton translocation; hence, it does not contribute to ATP synthesis. Studies focusing on plants show that two respiration pathways with different energy yields provide the ability to maintain the redox, carbon, and/or energy balance in response to changing demands (Sluse and Jarmuszkiewicz, 1998; Ribas-Carbo et al., 2005; Sieger et al., 2005; Smith et al., 2009; Cvetkovska and Vanlerberghe, 2012; Dahal and Vanlerberghe, 2017). In addition to this function, AOX also decreases the rate of mitochondrial ROS formation (Maxwell et al., 1999; Vishwakarma et al., 2015). In fungi, low copper availability was shown to be connected to impaired respiration (cytochrome c oxidase pathway) (Joseph-Horne et al., 2001), which generally leads to ROS accumulation in the mitochondria; thus, positive regulation of alternative oxidase may compensate for this loss and minimize ROS formation, which was recently demonstrated in *Paracoccidioides brasiliensis* (Petito et al., 2020) and in *C. albicans*, where copper starvation led to mitochondrial SOD1 repression and AOX induction enhanced cytochrome c oxidase activity (Broxton and Culotta, 2016). NDH-2 is a rotenone-insensitive nonproton pumping oxidoreductase that catalyzes a reaction similar to that of complex I, but in contrast to complex I, NDH-2 is not involved in the generation of membrane potential. NDH-2 was identified in plants, fungi, and bacteria as well as in some important parasitic protists, such as *Plasmodium falciparum* and *Toxoplasma gondii* (Marres et al., 1991; Yagi, 1991; Kerscher, 2000; Roberts et al., 2004; Lin et al., 2011). These two members belonging to the phylum Apicomplexa lack genes encoding canonical complex I and possess only homologs of NDH-2 instead (Fry and Beesley, 1991; Gardner et al., 2002; Uyemura et al., 2004). Altogether, NDH-2 is widely distributed in several human pathogens but not in humans themselves; thus, inhibitors of this enzyme could have clinical importance. Several studies have shown that NDH-2 provides a mechanism to remove excessive reducing power to balance the redox state of the cell (Luttik et al., 1998; Overkamp et al., 2000; Melo et al., 2004; Rasmusson et al., 2004). As mentioned above, branched mitochondrial ETC is activated in both studied amoebas upon copper limitation. In addition to AOX, whose activity is increased in both amoebas, NDH-2 is the most upregulated protein in copper-starved *N. fowleri*. Although we cannot conclude the direct consequences of NDH-2 induction for copper starvation in *N. fowleri*, we believe that further studying the exact mechanisms underlying the fascinating maintenance of the delicate balance between ATP production, ROS generation, and redox status in these microorganisms would be exciting.

### DJ-1

One of the proteins most affected by copper limitation in *N. gruberi* shows homology to proteins belonging to the DJ-1/ThiJ/PfpI superfamily. Members of this superfamily are present in many organisms from bacteria to humans, and the most studied is the human homolog due to its role in several diseases, such as autosomal recessive early-onset Parkinson’s disease (Bonifati et al., 2003). DJ-1 is also suggested to be one of the potential tumor markers and is strongly implicated in the pathogenesis of cancer (Nagakubo et al., 1997; Fan et al., 2015; Yu et al., 2017) and ischemia-reperfusion injury (Wang et al., 2017). Hundreds of publications explore the human homolog of DJ-1 and suggest many diverse functions, with roles as molecular chaperones (Cookson, 2003; Meulener et al., 2005), glyoxalases (Lee et al., 2003), proteases (Chen et al., 2010), and transcriptional regulators (Trempe and Fon, 2013), but the one function connecting these studies is the stress sensor reacting to oxidative stress and protecting cells from ROS (Taira et al., 2004; Inden et al., 2006). Some studies show that cells with a high level of DJ-1 are resistant to oxidative stress and neurotoxins, while lower levels of DJ-1 increase cells’ vulnerability to oxidative stress (Inden et al., 2011). Therefore, we assessed the abundance of NgDJ-1 in cells treated with the ROS-inducing agents rotenone and PEITC. However, the expected induction of NgDJ-1 by ROS was not observed; in fact, the protein was downregulated in cells with higher ROS levels.

Human DJ-1 is predominantly localized to the cytoplasm, but it has been reported to be translocated to the mitochondria and nucleus under oxidative stress and to protect cells from oxidative stress-induced cell death (Irrcher et al., 2010; Kim et al., 2012). On the other hand, some studies also show that DJ-1 may be localized to mitochondria even in the absence of oxidative stress, where it directly binds to a subunit of complex I and somehow maintains its activity, since knockdown of DJ-1 in cells decreased complex I activity (Hayashi et al., 2009; Mullett and Hinkle, 2011). A recent study also showed its connection to ATP synthase, where DJ-1 is required for the normal stoichiometry of ATP synthase and to facilitate positioning of the β subunit of ATP synthase to fully close the mitochondrial inner membrane leak (Chen et al., 2019). Interestingly, our results show exclusively mitochondrial localization of NgDJ-1 regardless of copper availability. Only a few studies claim a certain link between copper metabolism and the DJ-1 protein. In 2014, Stefania Girroto and others suggested a putative role of DJ-1 as a copper chaperone for superoxide dismutase (Girotto et al., 2014). Two novel copper-binding sites, one Cu(I) binding site per monomer involving the highly conserved Cys-106 and the second Cu(I) binding site shared between two monomers, were identified, and the kinetics and binding affinity of DJ-1 to copper ions were determined (Girotto et al., 2014). Since the conserved Cys-106 analog is also present in the sequence of NgDJ-1 and the levels of the protein are regulated by copper availability in the amoeba, we may speculate about its role in the copper homeostasis of *N. gruberi*. Because NgDJ-1 is localized only to mitochondria, it may act as a storage site for copper that can be later allocated to complex IV when copper availability is limited, which is somewhat analogous to the case of plastocyanin in *Chlamydomonas* (Kropat et al., 2015). We may also consider the role of this protein as a protein with chelating properties to prevent free copper accumulation, which can cause increased ROS production and lead to impaired respiration. Since the levels of NgDJ-1 are affected by both copper limitation and copper excess, NgDJ-1 may play multiple roles in the amoeba.

### Iron Uptake in *N. gruberi*

In our recent work, we showed that *N. fowleri* utilizes a reductive system of iron uptake, as described in the model *S. cerevisiae* (Arbon et al., 2020). This mechanism relies on the extracellular reduction of ferric ions from proteins, chelates, or other sources before their import into the cell. In yeast, the high-affinity ferrous-specific iron transport system is composed of multicopper oxidase FET3 and FTR1 permease; thus, copper availability is crucial for maintaining iron homeostasis (Askwith et al., 1994; Kaplan and O’Halloran, 1996; Stearman et al., 1996). Herein, we showed the same preference for the reduced form of iron in *N. gruberi*, but the main and surprising difference is that in contrast to *N. fowleri*, iron uptake efficiency in the nonpathogenic amoeba is decreased in copper-limited conditions, which corresponds to studies on *S. cerevisiae* (Dancis et al., 1994a) and on the model green algae *Chlamydomonas reinhardtii* (Herbik et al., 2002). An interesting question remains whether the pathogenic amoeba employs a copper-independent iron uptake mechanism or prioritizes extremely efficient copper delivery to this system in times of copper deprivation. The second hypothesis is rather unlikely since the *N. fowleri* iron uptake system has been previously shown to not be inducible even by iron starvation (Arbon et al., 2020).

### *N. gruberi* *and* *N. fowleri*: So Similar yet so Different. Similarities and Differences

**FIGURE 6:**
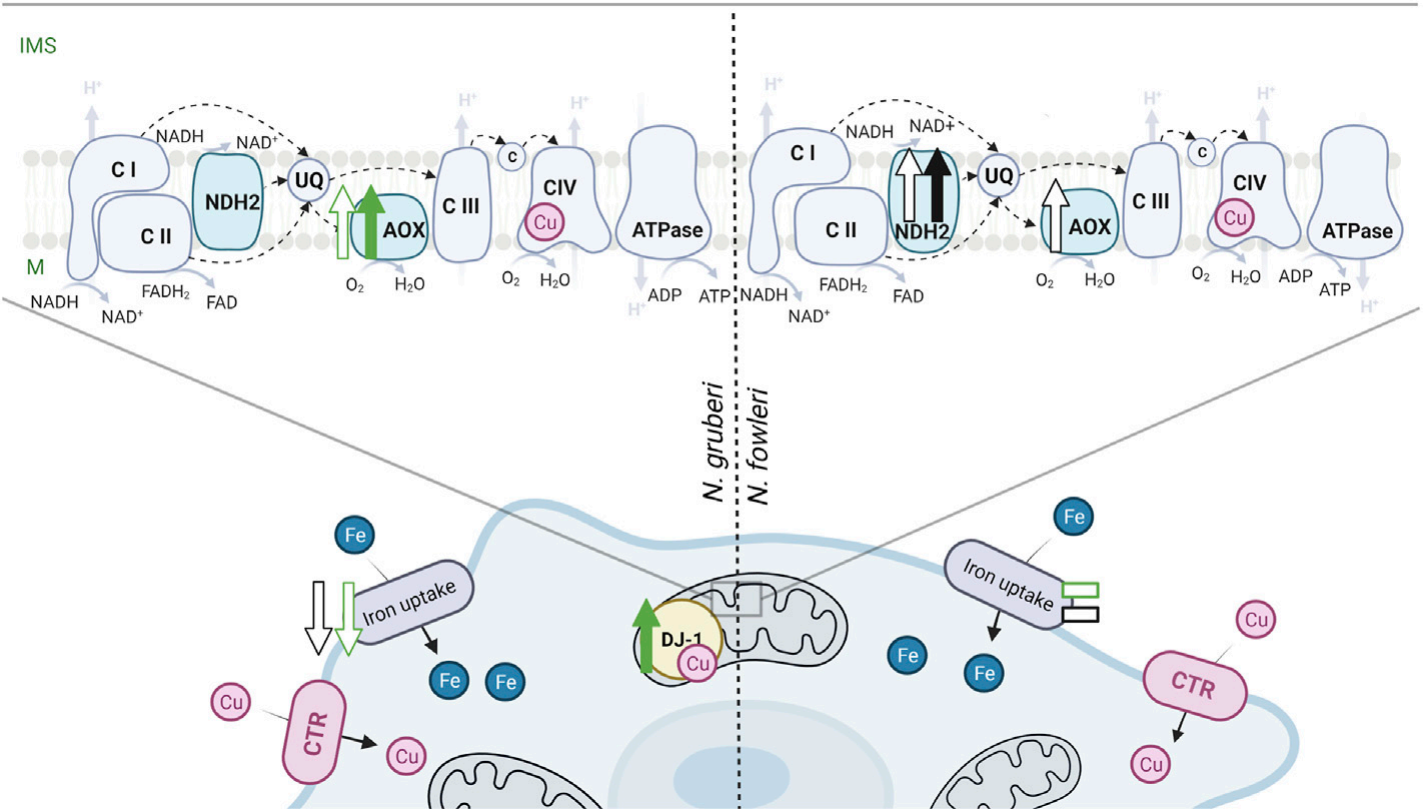
The main effect of copper deprivation on the cellular processes of *N. gruberi* and *N. fowleri*. The green color corresponds to the effect of the copper chelator BCS, and changes caused by neocuproine are depicted in black. Upward pointing arrows represent an increase in copper-deprived conditions, downward pointing arrows denote a decrease in copper-deprived conditions, and dashes indicate no copper-induced changes. Full arrows represent the results from the proteomic analysis, and empty arrows represent the measured activity. Respiration chain complexes are marked by appropriate numbers, UQ–ubichinol/ubichinone, c–cytochrome c. Created with BioRender.com.

Altogether, our study reveals how *N. gruberi* and *N. fowleri* deal with copper deprivation and highlights the differences between the two amoebas (Figure 6). We showed that while both amoebas use Ctr homologs to acquire copper and increase the activity of the branched mitochondrial ETC when copper is limited, their responses to copper limitation differ significantly. Although copper bioavailability limitation in the growth medium results in a more pronounced decrease in the cellular concentration of the metal in *N. fowleri* in comparison to *N. gruberi*, the growth of the pathogen is not affected, and the intracellular copper chelator neocuproine is required to observe a copper-related phenotype. Moreover, even when neocuproine was used to starve the cells for copper, iron uptake efficiency was not affected in *N. fowleri*, unlike *N. gruberi*. To hypothesize whether the particularities in *N. fowleri* copper homeostasis can contribute to its virulence would be an exaggeration, however, one must take into account the fact that copper is an important player in the host-pathogen relationship.

## Data Availability

The original contributions presented in the study are included in the article/Supplementary Material. The mass spectrometry proteomics data have been deposited to the ProteomeXchange Consortium via the PRIDE (Perez-Riverol et al., 2022) partner repository with the dataset identifier PXD032745, further inquiries can be directed to the corresponding author.

## Supplementary Material

Supplementary Table 1 List of whole-cell proteomes of both amoebas under copper-rich and copper-deprived conditions. The list is organized into thirteen sheets containing separately raw data of comparative proteomics results of *N. gruberi* (NG) or *N. fowleri (NF)* cells incubated in 25 μM BCS (BCS) or 5 μM neocuproine (NEO) compared to cells cultured in the presence of 25 μM Cu2SO4 (cu), sheets containing all upregulated (UP) or downregulated (DOWN) proteins under the indicated copper-depleted conditions and the list containing summarized table of significantly changed proteins of both amoebas in both copper-deprived conditions. Upregulated and downregulated proteins were selected and filtered based on the criteria listed in the Methods section.
