# Supplementary material for: Copper Metabolism in Naegleria gruberi and Its Deadly Relative Naegleria fowleri
Source: Front Cell Dev Biol. 2022 Apr 11;10:853463. doi: 10.3389/fcell.2022.853463 (PMC9035749; doi:10.3389/fcell.2022.853463)
Supplement: Supplementary file 1 [file DataSheet1.PDF]

Supplementary

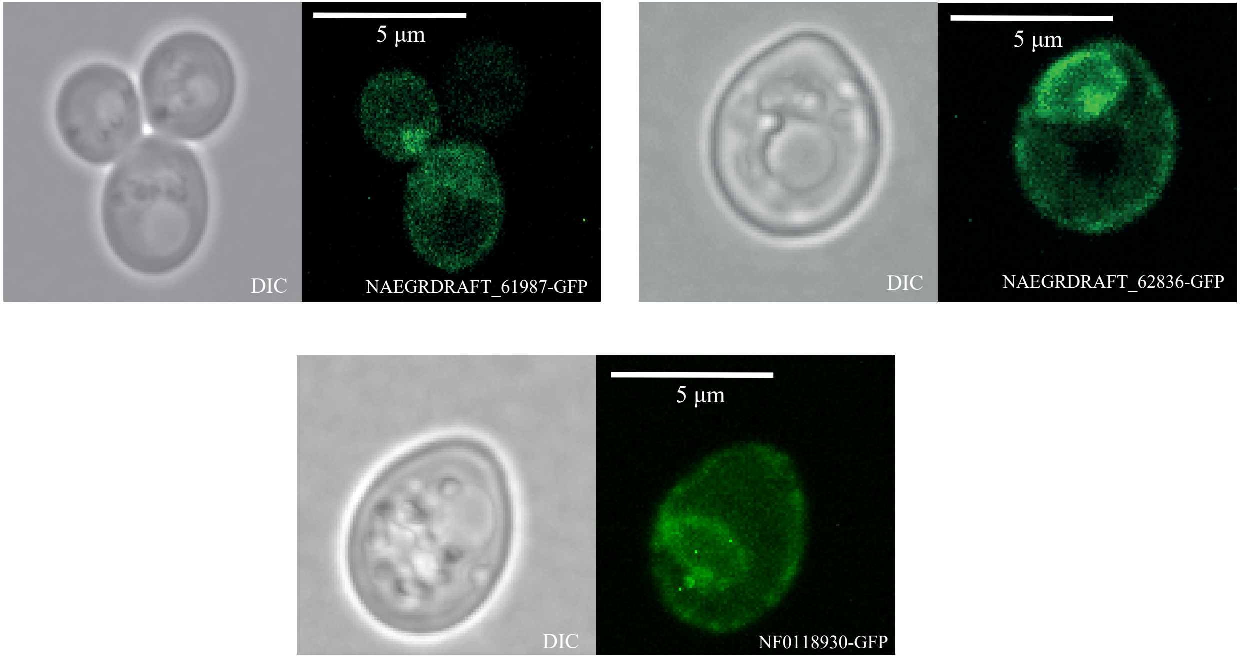

Figure S1. Localization of proteins homologous to yeast Ctrs by fluorescence microscopy.
Wild-type yeast BY4147 expressing putative copper transporters from *N. gruberi* (NAEGRDRAFT_61987 + pUG35; NAEGRDRAFT_62836 + pUG35) and *N. fowleri* (NF0078940 + pUG35) linked with GFP.

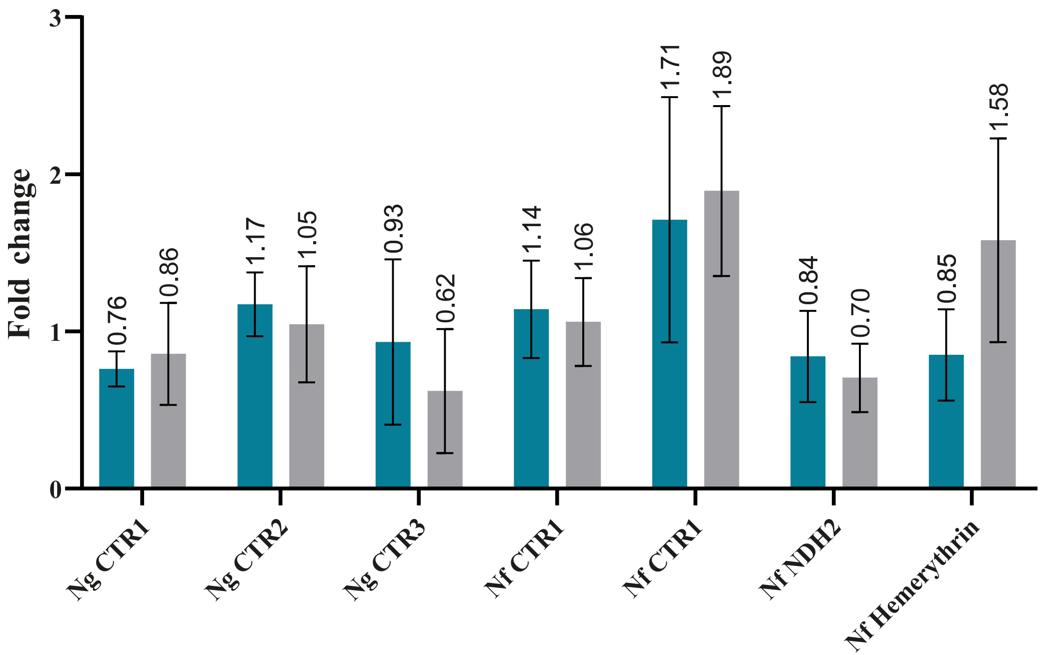

Figure S2. RT qPCR analysis of CTRs and selected genes encoding copper-regulated proteins of *N. gruberi* and *N. fowleri*. Visualization of fold change in transcript abundance of selected genes using copper-starved (25 µM BCS and 5 µM neocuproine) *N. fowleri* and *N. gruberi* cells (n=4).

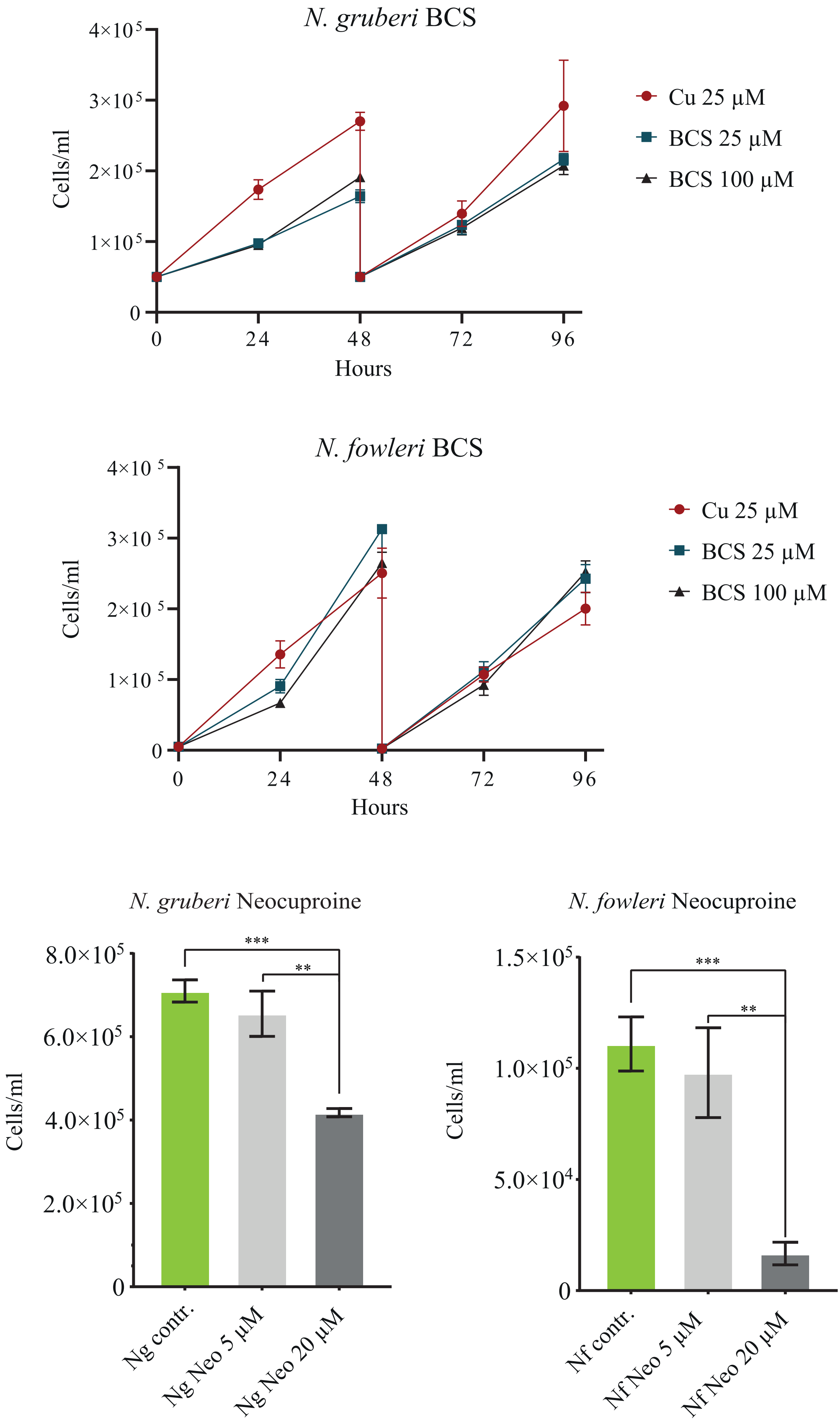

Figure S3. The effect of copper chelators BCS and neocuproine on the growth of *N. gruberi* and *N. fowleri*. The effect of extracellular chelator BCS was observed in long-term growth analysis with dilution of cells after 48 hours. *N. fowleri* and *N. gruberi* cells in growth medium supplemented with 25 µM Cu_2SO_4 (red circle) and 25 µM (blue square); 100 µM (black triangle) BCS. The effect of the intracellular chelator neocuproine was observed in one time point: 72 hours. *N. fowleri* and *N. gruberi* cells in growth medium supplemented with 5 µM (light gray) or 20 µM (dark gray) neocuproine. As a control, cells in a growth medium supplemented with DMSO were used (green). The average value of three biological replicates with relative standard deviation is shown.** indicates p-value ≤ 0.01; *** indicates p-value ≤ 0.001.

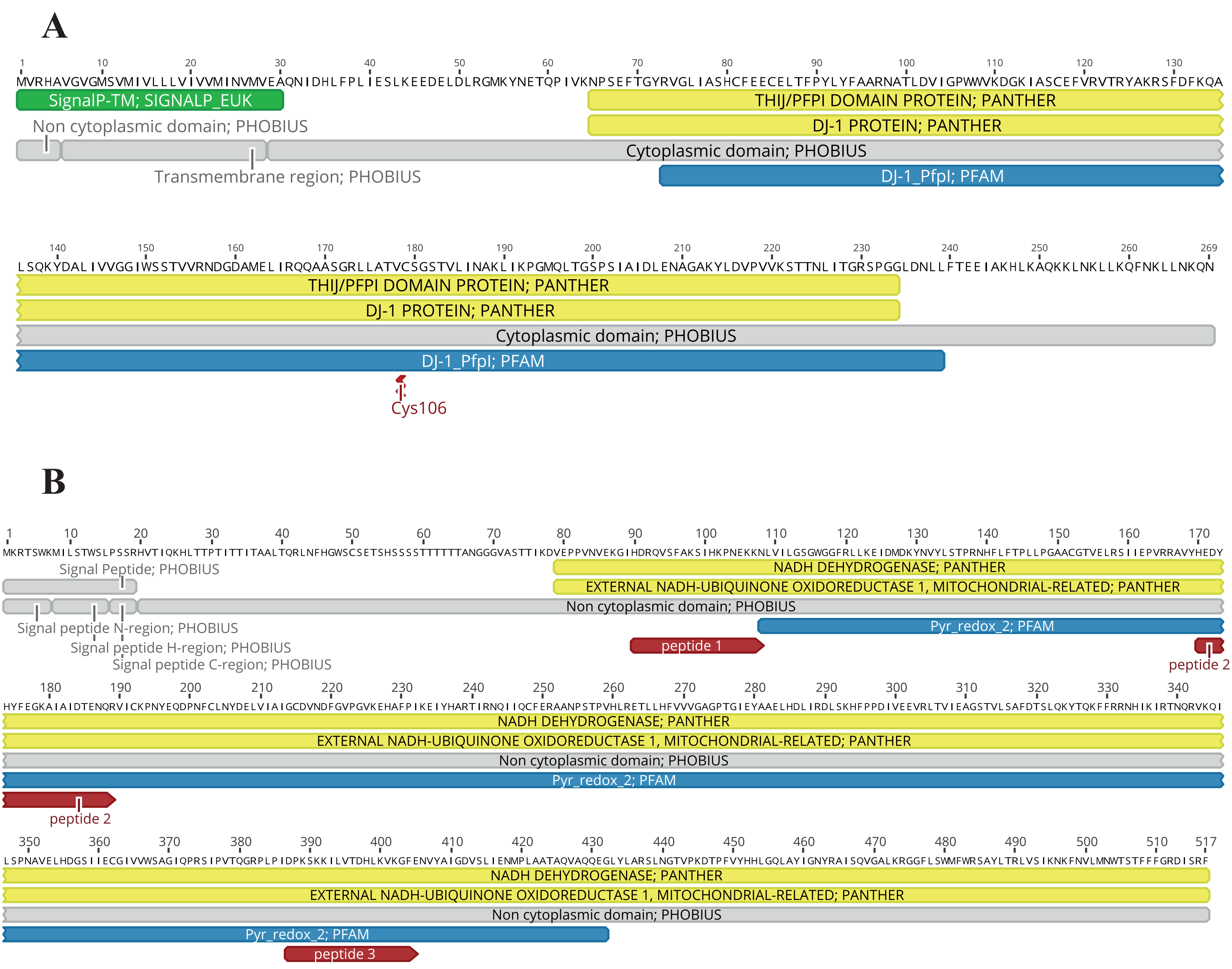

Figure S4. InterproScan analysis of protein sequence of NgDJ-1 (A) and NfNDH2 (B). Visualization of domains predicted by protein domain prediction software Phobius, Pfam, PANTHER, and SignalP 5.0 using InterProScan in Geneious Prime®. A) potential copper-binding site is marked in red; Cys 179 homologous to human Cys 108. B) Peptides used for antibody production are marked in red.

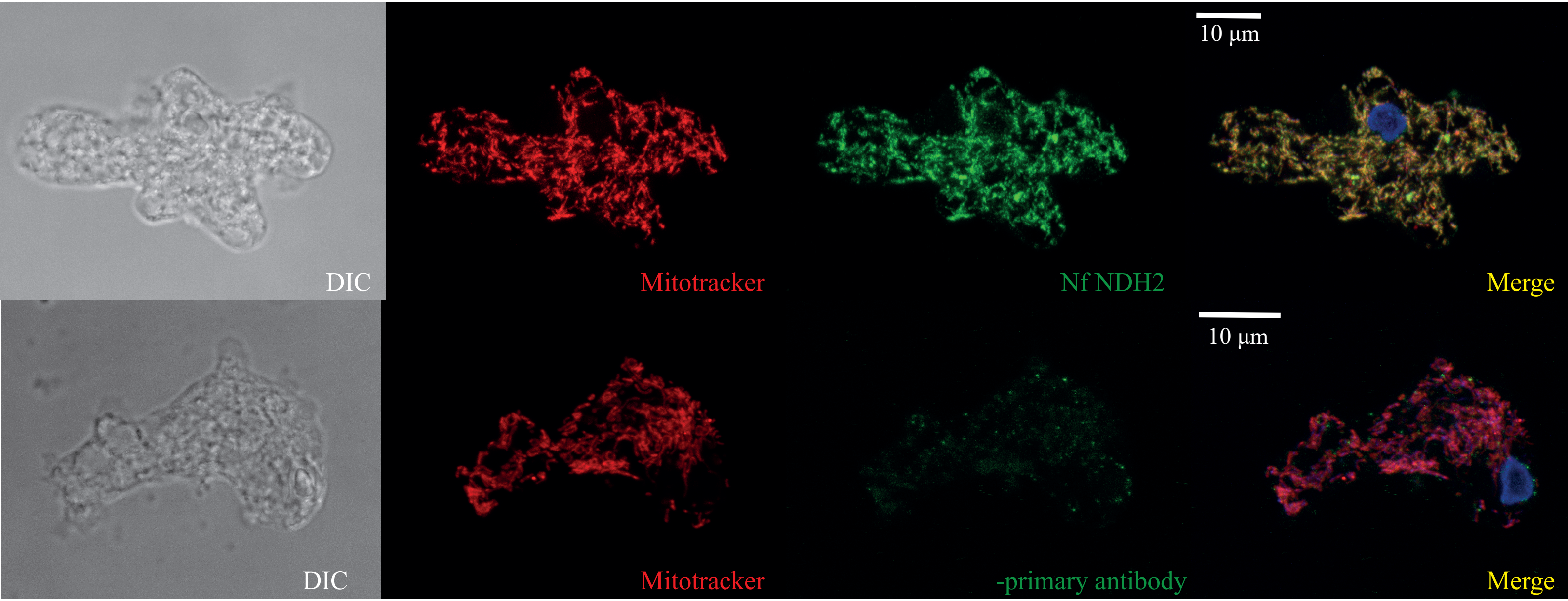

Figure S5. Localization of NfNDH2 by immunofluorescence microscopy using a polyclonal antibody (NfNDH2) on *N. fowleri* cells. DIC - differential interference contrast, MitoTracker was used to visualize mitochondria, and no addition of primary antibody was used as a control to show a potential background of fluorescence.

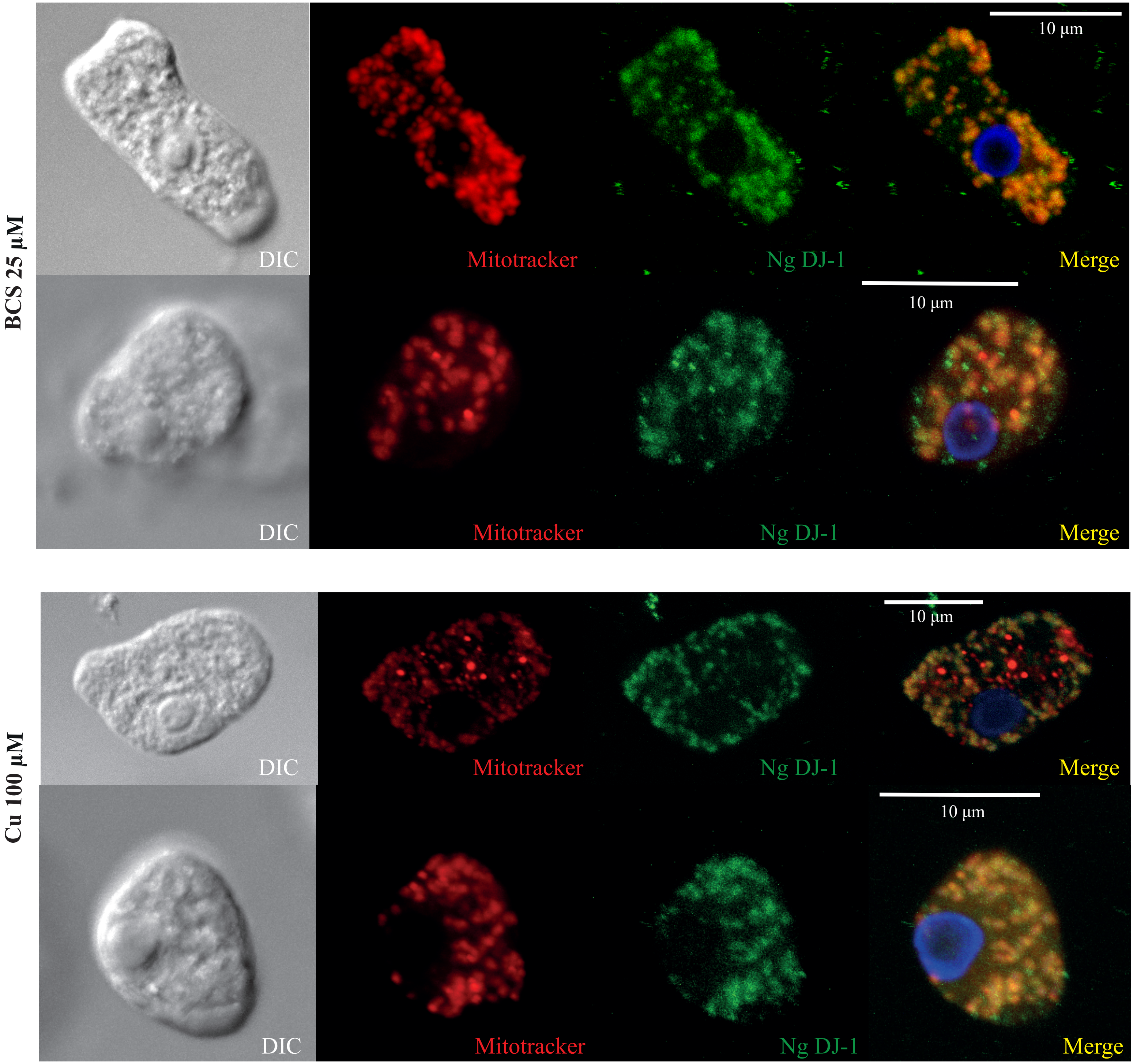

Figure S6. Localization of NgDJ-1 by immunofluorescencence microscopy using polyclonal antibody (NgDJ-1) on *N. gruberi* cells preincubated in low (BCS 25 µM) or high (100 µM Cu_2SO_4) copper availability.
DIC - differential interference contrast, MitoTracker was used to visualize mitochondria, and no addition of primary antibody was used as a control to show a potential background of fluorescence.
